# Exploring barriers to early breast examination and screening among Arab women in the MENA region: A KAP study

**DOI:** 10.1016/j.heliyon.2025.e42167

**Published:** 2025-01-23

**Authors:** Ehsan Qtaishat, Reem Al-Ajlouni, Khawlah Ammar, Mohammed Liswi, Abdallah Al-Ani, Rasha Fakheraldeen, Shorouq Al-hasson

**Affiliations:** a Jordan Breast Cancer Program, King Hussein Cancer Foundation, Amman, Jordan; b Office of Scientific Affairs and Research, King Hussein Cancer Center, Amman, Jordan

**Keywords:** Breast cancer, Arab, Screening, Early detection, Practice, Knowledge

## Abstract

**Aim:**

To examine the knowledge, attitudes, and practices (KAP) regarding early breast cancer screening among women across the MENA region.

**Methodology:**

This cross-sectional investigation deployed a survey designed to investigate women's KAP with regards to breast cancer signs and symptoms, early breast cancer detection methods, sources of knowledge, and barriers towards early detection exams. The survey was distributed over social media platforms during the period between June 2022–September 2022. Responses were reported as frequencies and analyzed per the participants demographic characteristics.

**Results:**

A total 2681 Arab women were included in the final analysis. Sudan (31.3 %), Saudi Arabia (15.6 %), and Palestine (14.0 %) were the most represented countries. Only 53.4 % of participants were able to recognize at least 5 signs and symptoms of breast cancer. While family history was the most reported risk factor for breast cancer (85.6 %), early onset of menarche (23.0 %) and late onset of menopause (24.0 %) were the least recognized. Participants were well aware of self-breast examination (SBE) with 72.0 % practicing it on regular basis. Conversely, while nearly half of the sample practices clinical breast examinations (CBE) or mammography (MM), less than 50 % were knowledgeable abouts their proper frequencies or suitable age. In terms of barriers, lack of current breast issues and lack of knowledge were the most commonly reported barriers to all three forms of early breast cancer detection methods. Univariate analysis demonstrated that regularly practicing SBE and CBE were associated with higher knowledge of breast cancer signs and symptoms (all p < 0.05). Also, older participants were more likely to be aware and be regularly compliant with SBE, CBE, and MM (all p < 0.001). Area of residence did not impact knowledge or practices of early breast cancer detection methods.

**Conclusion:**

Among our population of sampled adult Arab women, knowledge of breast cancer, its risk factors, and early detection methods are not satisfactory. Thus, we recommend increased awareness efforts and a profound exploration of the effectiveness of such interventions.

## 1 Introduction

Breast cancer is the most common type of cancer and cause of mortality among females worldwide [1]. The incidence and mortality of breast cancer are the highest among low-to-middle income countries and are on the rise in the Middle East and North Africa (MENA) region [2,3]. While over 90 % of breast lesions are benign, only 45 % of such diagnoses are symptomatic thus amplifying the role of early detection through screening [4]. Due to the lack of systematic registries, the exact epidemiology of breast cancer in the MENA region is hard to estimate [5]. Available data suggests that breast cancer comprises 14–42 % of all tumors diagnosed in Arab women [6]. The Global Burden of Disease estimates demonstrate a 377.9 % rise in incidence, a 203.7 % rise in deaths, and a 197.2 % rise in disability-adjusted life years across the MENA region from 1990 to 2019 [7]. This increase is believed to be partly a result of improved oncological care, implementation of screening programs, and better reporting.

Early breast cancer detection using early detection exams (EDEs), such as Self Breast Examination (SBE), Clinical Breast Examination (CBE), and Mammography (MM), is reported to improve prognosis and decrease both morbidity and mortality [8,9]. These techniques are both clinically effective and cost-effective even in low-to-middle income nations [10]. In fact, the disparities in breast cancer survival among developed and developing nations are partly attributed to constant improvements in screening techniques [11], in addition to improved management. Such variance in mortality, which can be observed between and within regions, is further amplified by differences in breast cancer awareness and access to screening [4]. It should be noted that patients with breast cancer in the MENA region, irrespective of economic status, present at a lower median age and with more advanced disease than their Western counterparts [12]; thus, regional guidelines must be set within the unique clinicopathological context of this affected population.

Perceived risk of cancer is impacted by individuals' beliefs about cancer and perceived susceptibility to cancer [13,14]. In fact, the Health Belief Model expands on such an ideology and proposes that perceived severity, susceptibility, benefits, and barriers are primary influencers of adopting health seeking behaviors [15,16]. In light of what's above, this study aims to examine breast cancer awareness, engagement in breast cancer early detection, and attitudes among Arab women.

## 2 Methodology

### 2.1 Setting, sampling and design

We have conducted a cross-sectional investigation of breast cancer early detection and screening and its associated practices among female participants. Our inclusion criteria include Arab, female-born, and adult participants between the ages of 18–69 years, currently residing within the MENA region. Participants younger or older than the aforementioned age bracket, failed to consent, or provided responses from outside the MENA region were excluded accordingly. We recruited participants using social media platforms from June 2022–September 2022. The study's survey was developed via Google Forms and uploaded to SurveyMonkey as the principal platform for data collection. Females within the aforementioned age group and are current residents of the following Arab countries (i.e., Sudan, Saudi Arabia, Palestine, Lebanon, Jordan, and United Arab Emirates) were targeted via social media advertisements and promotions, primarily Facebook.

### 2.2 Data collection instrument

We have developed a survey consisting of 3 distinct domains including I) demographics, II) knowledge of breast cancer, III) knowledge, practice and attitude of EDE. [**Refer to Additional file 1**]. The demographics section contained items pertaining to biological sex, marital status, area of residence, educational level, type of occupation, and family history of breast cancer among others. Several dimensions of breast cancer knowledge were examined including general facts about breast cancer, signs and symptoms, risk factors, optimal treatment, expected survival rates, and awareness of early detection methods. All the aforementioned items utilized a 5-point Likert scale (1 = strongly disagree, 2 = disagree, 3 = neutral, 4 = agree, 5 = strongly agree).

Domains III examined participants’ source of knowledge of EDE modality, most optimal age for modality usage, frequency, timing, current practice, barriers and facilitators to usage. The aforementioned items used either a multiple choice question (MCQ) or a dichotomous yes/no format. All items within the questionnaire and their corresponding themes were validated by a panel of breast cancer and public health experts for the aforementioned Arab countries. The data collection instrument was pilot tested on 25 participants removed from the final analysis.

### 2.3 Statistical analysis

All data was cleaned and analyzed using SPSS software 28, Categorical variables were presented as frequencies [n (%)], while continuous variables (e.g., knowledge score) were presented as means ± standard deviations. Categorical associations between different variables were examined using Chi-square. Mean differences in responses and domain scores were examined using ANOVA test. Knowledge scores were determined by calculating the average mean of all items constituting said domains and compared between categories using the aforementioned statistical tests. Multiple correction for multiple comparisons was done using the Bonferroni post-hoc method. All statistical tests are conducted with a 95 % confidence interval and a 5 % error margin. A p-value of less than 0.05 was considered statistically significant.

## 3 Results

### 3.1 Characteristics of respondents

Of 2985 women responding to the study's invitation, a total of 2887 agreed to participate in the study resulting in a response rate of 97 %. Of the included responses, 206 participants were excluded due to having a previous diagnosis of breast cancer; thus, the total number of women included within the final analysis was 2681. The greater portion of participants originated from Sudan (31.3 %), Saudi Arabia (15.6 %), and Palestine (14.0 %). The majority of participants were under the age of 40 (70.3 %), married (63.8 %), have higher education degrees (80.4 %), and are city dwellers (82.5 %). Table 1 demonstrates the sociodemographic characteristics of included participants.

**Table 1:** Sociodemographic characteristics of included participants.

**Variable**	**Frequency (n)**	**Percentage (%)**
**Country of origin**		
Sudan	839	31.3
Jordan	252	9.4
Saudi Arabia	419	15.6
United Arab Emirates	152	5.7
Lebanon	264	9.8
Palestine	376	14.0
Others	379	14.1
**Age Group**		
18–20	64	3.7
21–30	566	32.5
31–40	594	34.1
41–50	372	21.3
51–60	128	7.3
Above 60	20	1.1
**Marital status**		
Single	716	29.8
Married	1531	63.8
Widowed	43	1.8
Divorced	110	4.6
**Educational level**		
Elementary	105	4.4
High school	368	15.3
Higher education	1937	80.4
**Area of residence**		
Rural	421	17.5
Urban	1980	82.5

### 3.2 Knowledge of risk factors, signs, and symptoms

Of 2612, 78.7 % believed that breast cancer is the most common malignancy among females while only 5.6 % chose cervical cancer. When asked about breast cancer signs and symptoms, participants recognized presence of breast lumps (69.9 %), unusual nipple discharge (61.1 %), and armpit lumps (60.2 %), and change in size and shape of the breast (59.5 %) as the most likely signs and symptoms associated with breast cancer malignancy (Table 2). Out of the 11 presented signs and symptoms, 53.4 % were able to recognize at least 5 while 17.4 % recognized none.

**Table 2:** Knowledge of breast cancer signs and symptoms (N = 2681).

	**Agree**		**Disagree**	
**Signs and symptoms**	**Frequency (n)**	**Percentage (%)**	**Frequency (n)**	**Percentage (%)**
Presence of breast lump	1871	69.9	810	30.2
Color and temperature changes	113	41.5	1568	58.5
Skin changes	1059	39.5	1622	60.5
Nipple retraction	1294	48.3	1387	51.7
Itching	981	36.6	1700	63.4
Armpit lump	1614	60.2	1067	39.8
Skin thickness	1346	50.2	1335	49.8
Unusual pain in the armpit	1052	39.2	1629	60.8
Unusual pain in the breast	1001	37.3	1680	62.7
Change in size and shape of breast	1595	59.5	1086	40.5
Unusual nipple discharge	1639	61.1	1042	38.9

**Fig. 1:**
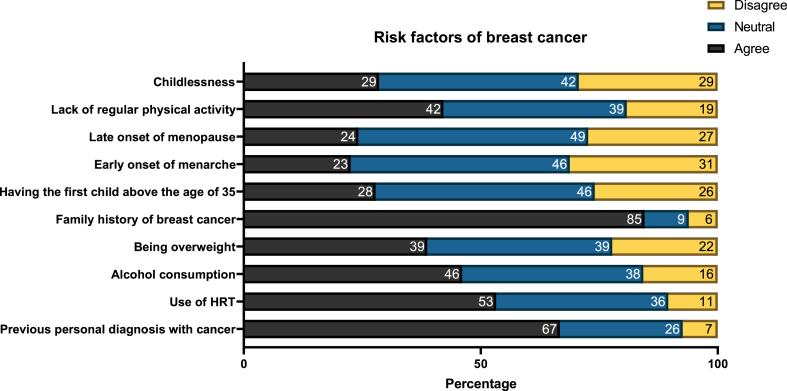
Participants' awareness of risk factors associated with developing breast cancer.

The most common recognized breast cancer risk factors among participants were family history of breast cancer (85.6 %), previous diagnosis with other than breast cancer (66.7 %), use of hormone replacement therapy (53.2 %), alcohol consumption (46.1 %), and lack of regular physical activity (42.1 %). On the other hand, factors such as late onset of menopause, early onset of menarche, having children after the age of 35, and childlessness were contested among participants (Fig. 1).

### 3.3 Knowledge and practices towards early detection exams (EDE)

Of 2596 respondents, 87 % were aware of self-breast examination (SBE). The majority of participants claimed that healthcare workers (HCWs) (42.5 %), social media (41.6 %), and awareness lectures (34 %) were their sources of awareness towards SBE. A total of 1354 participants reported on the most suitable age for starting SBE, of which only 26.4 % were able to recognize the correct answer. In terms of SBE's frequency, 64.8 % of 2211 respondents believe that ‘once a month’ is most appropriate. In terms of SBE's timing, the greater majority of 2209 participants believe that ‘1 week to 10 days after the onset of menstruation’ is most appropriate (47.4 %) while 25.4 % did not commit to any answer. Out of 2218 respondents, 72.0 % were practicing SBE on a regular basis.

Among 2543 respondents, 62 % were aware of clinical breast examination (CBE). The most common sources of information on CBE includes HCWs (40.2 %), awareness lectures (20.1 %), and social media (18.7 %). A total of 786 participants responded on the most suitable age for CBE, of which 12.8 % knew the correct answer. In terms of CBE's frequency, only 380 participants answered correctly—Once yearly starting at the age of 25— out of 1537 respondents (24.7 %). Out of 1159 respondents, 50 % reported being subjected to regular CBE.

Finally, 68 % of 2506 respondents were aware about mammograms (MM). The main source of information about MMs were HCWs (45.5 %), followed by social media (23.4 %). In terms of MM's frequency, 46.8 % of 1640 respondents knew that MMs should be conducted once yearly after the age of 40 years. With respect to practicing regular MMs, out of 764 respondents above the age of 39, only 57 % were committed to yearly mammograms.

### 3.4 Facilitators, barriers, and challenges towards EDE

Table 3 demonstrates the facilitators of EDE among the included sample. Among our included participants (n = 2512), the most common facilitators of SBE included self-reassurance (71.5 %), compliance with age-oriented health guidelines (24.6 %), having health concerns (18.5 %), and advice from HCWs (14.5 %). On the other hand, a total of 596 respondents reported on the barriers to SBE which commonly included lack of breast issues (26.8 %), lack of knowledge on conducting SBE (25 %), negligence (15.4 %), and lack of family history (11.7 %).

**Table 3:** Facilitators of EDE among Arab participants.

	**SBE (N = 2512)**		**CBE (N = 957)**		**MM (N = 428)**	
**Facilitators**	**Frequency (n)**	**Percentage (%)**	**Frequency (n)**	**Percentage (%)**	**Frequency (n)**	**Percentage (%)**
Self-reassurance	1117	71.5	441	57.6	160	37.2
Compliance with age-oriented guidelines	384	24.6	161	21.0	91	21.2
Having health concerns	289	18.5	0	0.0	0	0.0
Recommendation from a healthcare worker	282	18.0	134	17.5	81	18.8
Minimal effort or cost	226	14.5	0	0.0	0	0.0
Presence of symptoms	88	5.6	186	24.3	73	17.7
Recommendation from friends/family	86	5.5	25	3.3	14	3.0
I don't know	23	1.5	10	1.31	0	0.0
Family history of breast cancer	12	0.8	0	0.0	7	1.6
Other	5	0.3	0	0.0	2	1.0

Among 765 participants, the facilitators for CBE included self-reassurance (57.6 %), presence of symptoms (24.3 %), compliance with age-oriented health guidelines (21.0 %), and advice from HCWs (17.5 %). Conversely, 711 respondents reported the following barriers: lack of breast issues (37.3 %), lack of knowledge on CBE (13.2 %), being too young for being tested (12.9 %), lack of family history (11.3 %), and fear of outcome (6.8 %).

**Fig. 2:**
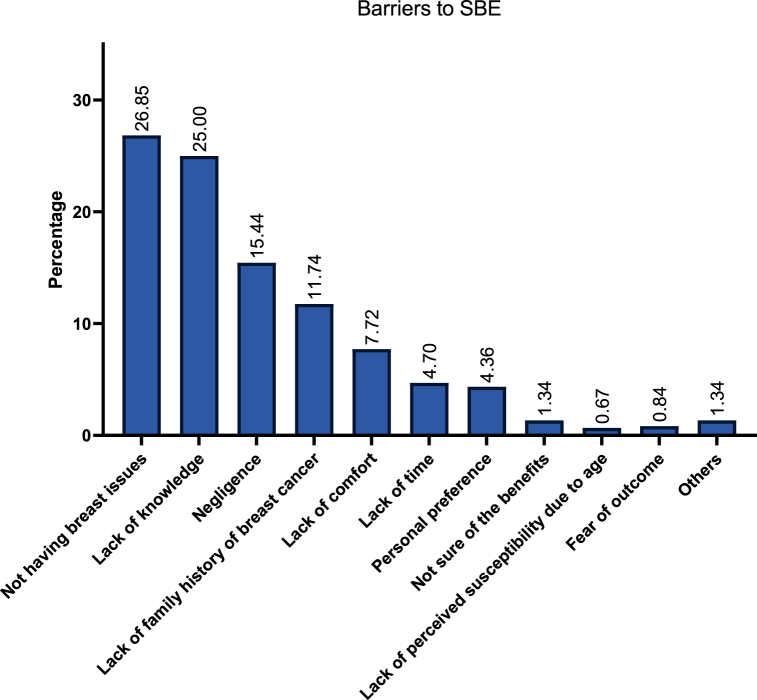
Participants' reported barriers to self-breast examination (SBE).

**Fig. 3:**
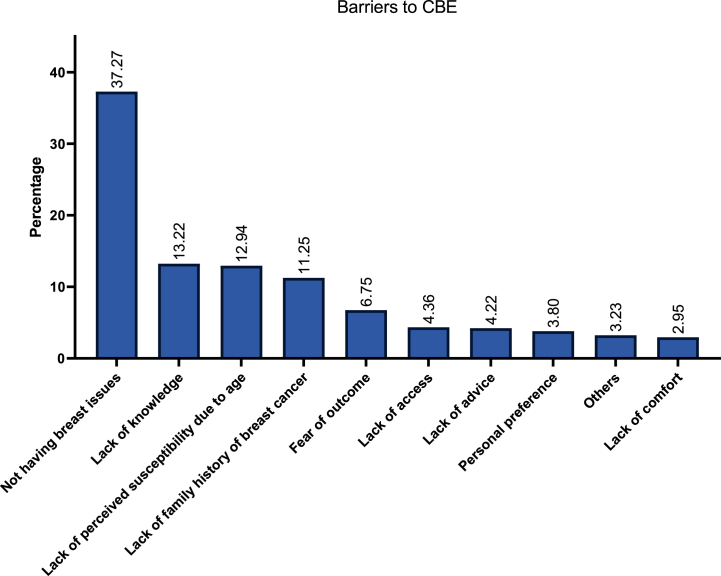
Participants' reported barriers to clinical breast examination (CBE).

**Fig. 4:**
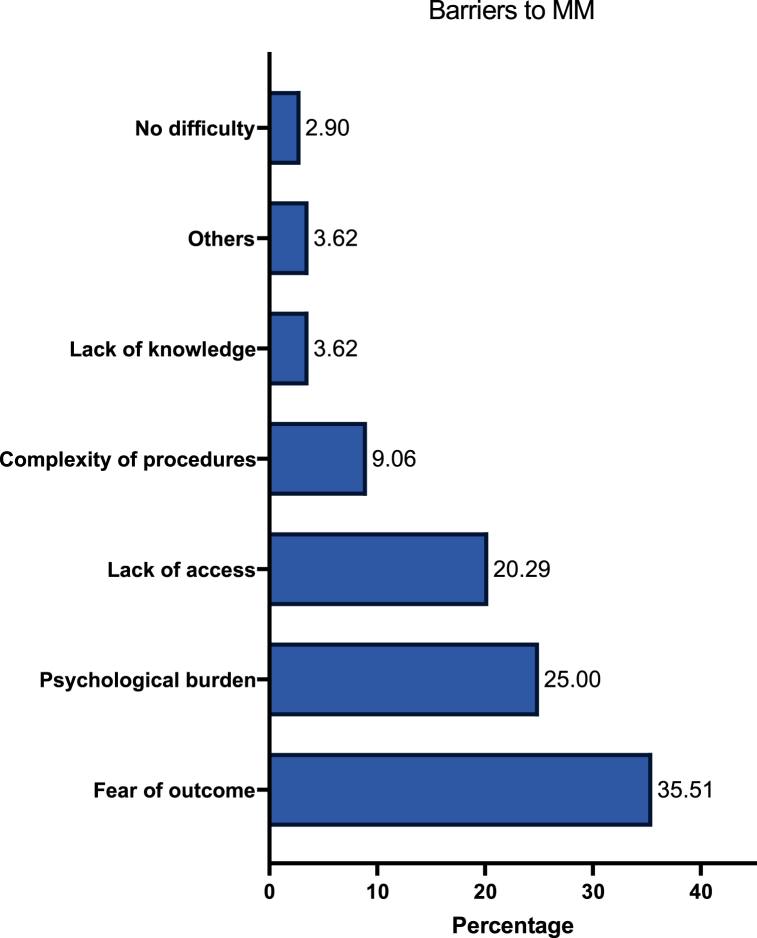
Reported barriers to mammograms.

Among 430 participants, factors promoting doing MMs included self-reassurance (37.2 %), compliance with age-oriented health guidelines (21.2 %), advice from HCWs (18.8 %), and presence of symptoms (17.0 %). A total 276 participants reported on the challenges faced during doing a MM. These primarily included fear of results (35.5 %), psychological anxiety and stress (25.0 %), and lack of access (20.3 %). Additionally, a total of 125 participants reported on the barriers to MM which commonly included lack of family history (28.0 %), lack of knowledge (19.2 %), lack of time (17.6 %), and lack of advice (12.0 %). Fig. 2, Fig. 3, Fig. 4 describe the barriers and challenges towards SBE, CBE, and MM, respectively. Moreover, across 1474 participants, increased awareness (100 %), lower costs (79.0 %), insurance coverage (54.7 %), MM-dedicated medical advice (52.6 %), and familial encouragement (45.6 %) were reported factors which may encourage women to pursue MMs.

### 3.5 Factors associated with practicing EDE and its associated knowledge

Univariate analysis demonstrated that respondents who regularly practice SBE have significantly higher knowledge of SBE (2.43 ± 0.82 vs. 1.99 ± 0.76; p < 0.001) and knowledge of breast cancer symptoms (6.54 ± 3.49 vs. 4.71 ± 3.46; p-value <0.001). Additionally, respondents who practice CBE have significantly higher knowledge of breast cancer symptoms (6.66 ± 3.48 vs. 6.18 ± 3.51; p = 0.007) but not CBE knowledge (1.40 ± 0.65 vs. 1.36 ± 0.66; p = 0.258). Finally, the practice of MM wasn't associated with significant differences in either breast cancer symptoms score (6.14 ± 3.70 vs. 5.89 ± 3.64; p = 0.350) nor MM knowledge score (1.60 ± 0.49 vs. 1.54 ± 0.50; p = 0.123).

Participants aware of SBE, CBE, and MM were significantly older than their unaware counterparts (all p-value <0.001) (**Refer to** Supplementary Table 1**; additional file 2**). Similarly, participants practicing SBE, CBE, and MM were significantly older than their non-practicing counterparts (all p-value <0.001). Neither educational level or area of residence affected practice of SBE, CBE, or MM with the exception of educational level and CBE practice (p = 0.027). Knowledge of risk factors score did not significantly differ among area of residence (p = 0.372) and age group (p = 0.559) (**Refer to** Supplementary Table 2; **additional file 2**). However, knowledge of risk factors score was significantly different among different countries (p = 0.046) with Sudanese participants scoring the highest while Lebanese participants scored the lowest.

Compared to their younger counterparts, participants in the above 39 years of age had significantly higher SBE (2.47 ± 0.81 vs. 2.34 ± 0.82; p = 0.009), CBE (1.46 ± 0.70 vs. 1.33 ± 0.61; p = 0.012), MM (1.57 ± 0.50 vs. 1.39 ± 0.49; p < 0.001), and total EDE knowledge score (3.60 ± 1.91 vs. 3.34 ± 1.90; p = 0.007). Significant differences among marital status were only found for CBE (Single: 1.47 ± 0.70; Married: 1.33 ± 0.62; Widowed: 1.31 ± 0.48; Divorced: 1.62 ± 0.82; p = 0.01) and MM knowledge scores (Single: 1.37 ± 0.48; Married: 1.51 ± 0.50; Widowed: 1.52 ± 0.51; Divorced: 1.40 ± 0.49; p < 0.001), whereby widowed participants had the highest MM score while also having the lowest CBE score. Furthermore, the aforementioned scores did not significantly differ per area of residence. Finally, participants with higher education degrees had a significantly higher total EDE knowledge score than their counterparts with lesser educational degrees (Elementary: 2.92 ± 1.69; High School: 3.08 ± 1.75; Higher Education: 3.39 ± 1.87; p = 0.007).

## 4 Discussion

Our study demonstrated that the knowledge of Arab women toward breast cancer's risk factors and signs and symptoms is subpar. Among the entire sample, only a handful of risk factors and signs were largely recognized. Additionally, while participants had acceptable rates of awareness of the different modalities of EDE, their knowledge of its timing and frequency was poor. Nonetheless, the rates for practicing SBE, CBE, and MMs were promising with at least half the sample committing to practicing different EDE modalities. In terms of facilitators for EDE practice, self-assurance, followed by the presence of symptoms and compliance with age-related screening guidelines, were the most commonly reported. Conversely, lack of breast issues and lack of knowledge were the most commonly reported barriers to pursuing EDE. Finally, the awareness and practice of EDE were associated with older age but not educational level or area of residence.

While concerning, the aforementioned results, particularly regarding the lack of proper levels of breast cancer knowledge are well-echoed within the Arab literature [[17], [18], [19], [20], [21], [22], [23], [24]]. On both a global and regional scale, awareness towards breast cancer is highly variable being highest for developed countries while reaching extremely low levels among developing nations [4]. This can be attributed to two factors: first, the differences in allocated resources towards breast cancer awareness and early screening. Developed nations such as the United States, United Kingdom, and Australia implement national screening programs and produce screening guidelines that fit the clinical and sociodemographic context of their respective populations [4]. On the other hand, developing nations, in addition to their limited public funds, often resort to SBE as a screening strategy and ultrasound as a diagnostic tool rather than mammography [25]. Second, the reason for inter-regional variation could be simply attributed to the methodological differences among studies in terms of target population characteristics, utilized data collection tools, and sampling strategies.

Within the MENA region, there is a dire need for the establishment and improvement of breast cancer awareness and screening programs [26]. Such is the case due to the lack of any reliable measures within the MENA region as well as in other low-to-middle income countries. This was further illuminated upon at the 12th Breast, Gynecological & Immuno-oncology International Cancer Conference of 2020, in which there was a unanimous consensus among both regional and international experts that the MENA region requires breast cancer awareness campaigns and screening programs that are in line with the latest WHO recommendations and are tailored per the socioeconomic characteristics of different affected nations [12].

Among our included sample of Arab women, the awareness of different EDEs was always higher than rates of practice. Moreover, this high level of awareness contrasted with the low rates of knowledge regarding the timing and frequency of those EDEs. While this may indicate that the reach of information regarding these services or the services themselves, in the case of MM, is adequate. However, it also shows that participants lack a concrete understanding of these different examinations. In the case of MM, the American College of Obstetricians and Gynecologists and the American Cancer Society recommend yearly mammograms starting at the age of 40 [27]. This particular piece of vital information was known correctly by less than half of the included sample. In addition, a significant portion of our included sample are motivated to pursue CBE and MMs due to the presence of symptoms; thus, demonstrating a deficiency in their appreciation of the purpose of EDEs, that is the early detection of cancer and not diagnosis.

Conquering the lack of optimal awareness towards breast cancer and its EDEs is of vital importance as it is associated with better uptake of screening services, higher levels of SBE practice and proficiency, and reduced psychological burden from fear of disease [20,28,29]. Interestingly, while all participants were exclusively sampled from social media platforms, their most commonly reported source of information on EDEs was HCWs. This further accentuates the role of primary caregivers in teaching women about EDEs, the normal structure of their breast, and on how to identify and report breast abnormalities [12,28].

The most common barriers to adopting breast cancer screening measures were perceived lack of breast issues and lack of knowledge. While the latter is considered a cornerstone barrier within the literature [30], the earlier is also well documented. A systematic review of breast cancer screening knowledge among studies originating from Singapore demonstrated that women expressed false perceptions with regard to avoiding breast cancer screening such as “being healthy” or “one would only get the cancer if they are looking for it” [31]. These beliefs may have a cultural component and must be targeted by HCWs within their awareness interventions. It appears that such barriers are consistently reported within Muslim countries, which further strengthens the presence of a cultural component to avoiding breast cancer screening [32].

Across the literature, various factors were associated with breast cancer screening knowledge and practice. Such factors include age, family history, educational level, occupation, area of residence and having children [9,33]. However, some factors, such as age and educational level, are contested within the literature [34,35]. Our results fall with the latter as only age was associated with significant differences in knowledge or practice.

### 4.1 Recommendations

Considering the aforementioned, MENA nations, particularly those with limited resources, should adopt holistic plans for establishing and improving breast cancer screening awareness. First, an investigation of primary HCWs abilities, knowledge, and practices with regards to breast cancer screening must be implemented. Second, awareness campaigns should be designed around the following: progression and development of breast cancer, risk factors, signs and symptoms, optimal and effective screening methods, and pursuing screening even in the absence of symptoms. Third, examining the impact, facilitators, and barriers of such plans within a sociocultural context unique to targeted populations. Fourth, ensuring that the aforementioned solutions could reach all age groups, educational levels, and areas of residence. Fifth, an in-depth exploration of the public perceptions of breast cancer, preferably through the Health Belief Model, should be conducted as a means to complement studies concerned with merely factual knowledge of breast cancer. Sixth, all the aforementioned should complement an already functional spectrum of breast cancer diagnosis, management, and research.

### 4.2 Limitations

Our results should be interpreted within the context of a number of limitations. The study adopted a cross-sectional design which may predispose the sampling frame to a snap-shot of the studied populations. The sampling through social media has its own set of biases as it may overestimate certain subsets of the Arab population, primarily those with internet access and interest in breast cancer online, which dominates the study. This is exemplified in the over-representation of well-educated participants; therefore, our results may not be generalizable at the level of the entire Arab peninsula but rather subsets with internet access and above average socio-economic living standards.

In terms of the data collection tool, the close-ended nature of the questionnaire could limit the range of choices presented to participants. Moreover, due to the fact that not all questionnaire items were mandatory, missing responses could have reduced the statistical power of the analysis or impacted the representativeness of the included sample. Finally, due to resource limitations, an optimal sample size for each included Arab nation was not reached, which may have impacted the rigor of our analysis and prevented us from making comparisons between participants from different Arab nations.

## 5 Conclusion

Knowledge of breast cancer, its risk factors, and EDE is below average for Arab women. This finding suggests that further awareness efforts and further exploration of breast cancer screening among Arab women are needed.

## CRediT authorship contribution statement

**Ehsan Qtaishat:** Writing – original draft, Methodology, Conceptualization. **Reem Al-Ajlouni:** Writing – original draft, Supervision, Conceptualization. **Khawlah Ammar:** Writing – review & editing, Writing – original draft, Supervision, Methodology, Formal analysis, Conceptualization. **Mohammed Liswi:** Writing – original draft. **Abdallah Al-Ani:** Writing – review & editing, Writing – original draft, Formal analysis. **Rasha Fakheraldeen:** Writing – original draft. **Shorouq Al-hasson:** Writing – original draft.

## Data availability statement

All data/data sets associated with this project can be requested from the corresponding author at a reasonable request.

## Ethical considerations

This study was approved by the KHCC Institutional Review Board (Approval #22KHCC39). All methods were out in accordance with guidelines and regulations of the aforementioned bodies. All participants read and signed a written informed consent form before continuing to complete the questionnaire. The informed consent form included the participants’ right to anonymity, confidentiality of their data, right to leave the study, and reassurance that their participation is completely voluntary, is not associated with any kind of short-term benefit or rewards.

## Consent for publication

Not applicable.

## Declaration of generative AI and AI-assisted technologies in the writing process

This manuscript did not utilize any form of generative AI or AI-assisted technologies in its writing or analyses processes.

## Funding

This research was funded by an intramural grant from the King Hussein Cancer Foundation.

## Declaration of competing interest

The authors declare that they have no known competing financial interests or personal relationships that could have appeared to influence the work reported in this paper.
