# Supplementary material for: Exploring barriers to early breast examination and screening among Arab women in the MENA region: A KAP study
Source: Heliyon. 2025 Jan 23;11(3):e42167. doi: 10.1016/j.heliyon.2025.e42167 (PMC11815657; doi:10.1016/j.heliyon.2025.e42167)
Supplement: Multimedia component 1 [file mmc1.pdf]

Supplementary Material

Additional File 1

Exploring barriers to early breast examination and screening among Arab women in the MENA region: A KAP study

Questionnaire v1.0

1. Do you agree to participate in the study?
    a. Yes
    b. No
2. Have you been previously diagnosed with breast cancer?
    a. Yes
    b. No
3. Country of origin
    a. Jordan
    b. Palestine
    c. Lebanon
    d. Egypt
    e. Sudan
    f. United Arab Emirates
    g. Saudi Arabia
    h. Other
4. Specify your biological sex
    a. Male
    b. Female
5. Marital status
    a. Single
    b. Married
    c. Divorced
    d. Widowed
6. Number of pregnancies
7. Age at first newborn
8. Age
9. Age at first menstrual cycle
10. Weight
11. Height
12. Do you have a family history of breast cancer?
    a. Mother
    b. Father
    c. Sister
    d. Brother
    e. Son
    f. Daughter
13. Have you ever consumed OCPs for more than 5 consecutive years?
    a. Yes
    b. No
14. Area of residence
    a. Urban
    b. Rural
15. Highest educational degree

    a. Illiterate
    b. Literate with no formal education
    c. Primary school
    d. Middle school
    e. High school diploma
    f. University Bachelors
    g. University Master's
    h. University PhD
    i. I prefer not to answer
16. Occupational sector
    a. Public
    b. Private
    c. Military
    d. Civil societies
17. Occupation
    a. Student
    b. Current worker
    c. Retired
    d. Housewife
    e. No current work
18. Do you have medical insurance?
    a. Yes, covers all illnesses
    b. Yes, covers some illnesses
    c. Yes, covers only cancer
    d. No
19. Of the following, which activity do you regularly participate in?
    a. Smoking
    b. Eating healthy foods
    c. Sleeping for at least 8 hours
    d. Playing sports
    e. Breast feeding for at least 6 months
    f. Weight control and monitoring
    g. Attending screening clinics periodically
    h. Eating vegetables on daily basis
    i. Eating moderate amounts of salt or sugar
    j. Being always anxious
20. When thinking about diseases that may affect women, which of the following encourages you to monitor your health
    a. Diabetes
    b. Hypertension
    c. Heart disease
    d. Breast cancer
    e. Cervical cancer
    f. Others
21. Which of the following cancers do you believe is the most prevalent among women?
    a. Breast
    b. Cervical
    c. Ovarian
    d. Leukemia
    e. Lymphoma

f. Uterine
g. Colon
h. Brain/CNS
i. Liver
j. Thyroid
k. Lung
l. Other

22. Which of the following cancers do you believe is the least dangerous to women
    a. Breast
    b. Cervical
    c. Ovarian
    d. Leukemia
    e. Lymphoma
    f. Uterine
    g. Colon
    h. Brain/CNS
    i. Liver
    j. Thyroid
    k. Lung
    l. Other
23. To what degree do you agree with the following statements (1 = Strongly disagree, 2 = somewhat disagree, 3 = neutral, 4 = somewhat agree, 5 = strongly agree, 9 = I don't know)

No.	Statement
1.	Breast cancer is the most prevalent cancer in Jordan
2.	Only women are afflicted with breast cancer
3.	Breast cancer can be detected during its early stages
4.	Breast cancer is contagious
5	Herbal medicine can be beneficial for breast cancer
6	Eating healthy food could fortify one's body against breast cancer
7	Playing sports could fortify one's body against breast cancer
8	Women with smaller breasts have a lesser chance of having breast cancer
9	Caffeine may cause breast cancer
10	Deodorants may cause breast cancer
11	Risk of breast cancer increases with age
12	Early detection of breast cancer is associated with better survival
13	90% of women with early detected breast cancer survive

24. According to your knowledge, which of the following signs and symptoms could indicate breast cancer?
    a. Presence of breast lump
    b. Color and temperature changes
    c. Skin changes
    d. Nipple retraction
    e. Itching
    f. Armpit lump
    g. Skin thickness
    h. Unusual pain in the armpit
    i. Unusual pain in the breast

   j. Change in size and shape of breast
   k. Unusual nipple discharge
   l. Other
25. What are the risk factors for developing breast cancer?
   a. Increasing age
   b. Alcohol consumption
   c. OCPs
   d. Early onset of menarche
   e. Eating high calorie/fatty foods
   f. Family history of cancer
   g. Having a first child after the age of 35
   h. Change of hormones
   i. Supplementary hormones for menopause
   j. Supplementary hormones for other diseases
   k. Lack of physical activity
   l. Late onset of menopause
   m. Obesity
   n. Not breastfeeding
   o. Personal history of cancer
   p. Radiation
   q. Smoking
   r. Stress
   s. Others
   t. I don’t know
26. According to your knowledge, what is the survival percentage of breast cancer in its early stages?
   a. 0%
   b. 1 – 10%
   c. 11 – 49%
   d. 50%
   e. 51 – 89%
   f. 90 – 100%
27. According to your knowledge, what is the survival percentage of breast cancer in its late stages?
   a. 0%
   b. 1 – 10%
   c. 11 – 49%
   d. 50%
   e. 51 – 89%
   f. 90 – 100%
28. According to your knowledge, what are the treatment modalities for breast cancer in its early stages?
   a. Chemotherapy
   b. Radiation therapy
   c. Surgical removal
   d. Others
   e. No treatment
   f. I don’t know
29. According to your knowledge, what are the treatment modalities for breast cancer in its late stages?
   a. Chemotherapy

b. Radiation therapy
c. Surgical removal
d. Others
e. No treatment
f. I don’t know

30. With regards to early breast cancer screening, which of the following diagnostic modalities are you familiar with?
    a. Self breast examination
    b. Clinical breast examination
    c. Mammogram
    d. Ultrasound
    e. Biopsy
    f. Others
    g. I don’t know

Self breast examination (SBE)

1. How did you know about SBE
    a. Doctor
    b. Family or friends
    c. School or university
    d. Educational video on the internet
    e. Social media
    f. Awareness lectures
    g. Booklets
    h. Others
2. At what age should you start SBE
3. What is the frequency of doing SBE
    a. Once daily
    b. Once weekly
    c. Once monthly
    d. Once yearly
    e. I don’t know
    f. Only when symptoms are present
4. What is the most suitable time for SBE
    a. During menstruation
    b. After 7 to 10 days from start of menstruation
    c. Before 7 to 10 days from start of menstruation
    d. At whatever time
    e. I don’t know
    f. Other
5. Do you practice SBE
6. Have you ever recognized anything unusual during SBE
    a. Thick skin
    b. Presence of a lump
    c. Inverted nipple
    d. Unusual discharge
    e. Itchy feeling
    f. Change in breast depth
    g. Change in breast shape or size
    h. Wrinkles in breast
    i. Change in color or temperature of breast

    j. Others
7. How did you react to you noticing an unusual breast symptom
    a. Visited a doctor
    b. Took advice from friends and family
    c. Searched on the internet
    d. Nothing
    e. Others
8. What are your reasons for conducting SBE
    a. For reassurance
    b. I reached the age by which this test is appropriate
    c. It doesn't need effort or cost anything
    d. It doesn't need a doctor or specialist
    e. Presence of symptoms
    f. Advice from doctor
    g. Advice from close members
    h. Booklets
    i. Others
9. Why don't you conduct SBE
    a. I don't have breast issues
    b. I don't think SBE is necessary
    c. I don't feel comfortable
    d. I don't know how
    e. I don't have time
    f. Its not acceptable in the family
    g. I am shy
    h. I forget
    i. It's a burden to do it monthly
    j. I don't this its necessary
    k. I don't have a family history of cancer
    l. Others

Clinical breast examination (CBE)

1. How do recognize CBE
    a. Yes
    b. No
2. How did you learn about CBE
    a. Doctor
    b. Family or Friends
    c. School or University
    d. Media (Specify)
    e. Social Media (Specify)
    f. Lectures
    g. Brochures
    h. JBCP lectures
    i. Others, please specify
3. At what age should CBE be started?
4. Have you ever had CBE?
5. How many times should you conduct CBE
    a. Once annually after the age of 25
    b. Annually after the age of 40
    c. Annually irrespective of age

    d. Once per lifetime
    e. I don’t know
    f. Others
6. What are your reasons for conducting SBE
    a. For reassurance
    b. I reached the age by which this test is appropriate
    c. Presence of symptoms
    d. Advice from doctor
    e. Advice from close members
    f. Booklets
    g. Others
7. What were the doctor’s recommendations after your last CBE
    a. Conduct mammogram
    b. Conduct ultrasound
    c. Conduct a biopsy
    d. Conduct a surgical excision
    e. Nothing
    f. Monthly visit
    g. Visit another doctor of a different specialty
    h. Repeat CBE and SBE
    i. Others
8. Why don’t you conduct CBE
    a. High costs
    b. Hard to find an appointment
    c. Lack of time
    d. Fear of result
    e. Too young for CBE
    f. No breast symptom
    g. Didn’t get an advice to do it
    h. I am shy to visit a doctor
    i. Visiting a doctor doesn’t help
    j. Fate
    k. Lack of support from spouse
    l. Lack of support from family
    m. Others

Mammogram (MM)

1. How do you learn about MM
    a. Doctor
    b. Family or friends
    c. University
    d. Lecture at work
    e. Others
2. Have you ever conducted a MM
3. Why do women conduct MM
    a. After a doctor’s advice
    b. Annually after the age of 40
    c. After presence of symptoms
    d. Others
4. What is the most appropriate age for MM
    a. Before the age of 40

    b. Starting from the age of 50
    c. Other
    d. I don’t know
5. At what frequency should a woman do the mammogram?
    a. Yearly
    b. Every two years
    c. Every 4 years
    d. Never
    e. I do not know
    f. Others, specify
6. How many times did you do mammogram in the last five years?
    a. Never
    b. Once
    c. 2-5 times
    d. More than 5 times
    e. I don't know
7. In the past 12 months, have you done Mammogram examination?
    a. Yes
    b. No
8. What were he reasons which made do the Mammogram examination the last time?
    a. Just a check up
    b. Reached an age where I am more vulnerable to breast cancer
    c. Doesn’t require going to a physician
    d. Symptoms appeared
    e. Recommendation from doctor
    f. Recommendation from people surrounding me
    g. School or University
    h. Media
    i. Lectures
    j. Brochures
    k. JBCP lectures
    l. JBCP Twitter
    m. JBCP Facebook
    n. JBCP YouTube
    o. Follow up
    p. Others (Specify)
9. In which institutions you can do the Mammography in?
    a. MOH Public Health Centers
    b. Private Health Centers / Radiology
    c. Public Hospitals
    d. Military Hospitals
    e. Private Hospitals
    f. King Hussein Cancer Center
    g. Others (Specify)
10. Please tell me what challenges you faced in conducting the Mammography? What else?
    a. Expensive
    b. Husband / family opposed
    c. It takes time
    d. Time limitations

e. It is not useful in detecting Cancer
f. Knowing which exam to do
g. Knowing how to do the exam
h. Awareness of screening centers' locations and service availability
i. Fear of results
j. No close health care center which provides Mammography
k. Painful
l. Difficult procedures at the exam centers
m. Others (specify)

11. What were the reasons which prevented you from doing the mammography? What else?
    a. Expensive
    b. Husband / family opposed
    c. It takes time
    d. Time limitations
    e. It is not useful in detecting Cancer
    f. Knowing which exam to do
    g. Knowing how to do the exam
    h. Awareness of screening centers
    i. Fear of results
    j. No close health care center which provides Mammography
    k. Painful
    l. I have other financial priorities
    m. A woman in my age should not be conducting mammogram screening
    n. No symptoms appeared
    o. I don't know anything about it
    p. Others (specify)
12. Has your doctor advised you to do the early screening for breast cancer on a regular basis just for checkup?
    a. Yes, Self-Breast Examination
    b. Yes, Clinical Breast examination by doctor
    c. Yes, Mammography
    d. Others (specify)
    e. No, he did not advise
13. If a woman suspects that they have breast cancer, which physician should they consult?
    a. Surgeon
    b. Internist
    c. Gynecologist
    d. Family Medicine
    e. Early detection clinics
    f. Other, please specify
    g. I don't know
14. To what degree do you agree with the following statements (1 = Strongly disagree, 2 = somewhat disagree, 3 = neutral, 4 = somewhat agree, 5 = strongly agree, 9 = I don't know)

No.	Statement
1.	Self-examination cannot detect the presence of breast cancer
2.	Most women know how to perform breast self-examination

3.	It is important to raise awareness among females of all ages about the importance of early detection screenings for breast cancer.
4.	Self-examination for breast cancer is not sufficient
5	Breast self-examination is important for early detection of breast cancer
6	Clinical breast examination alone is not sufficient for detecting breast cancer
7	Clinical breast examination is important in cases where there are palpable masses
8	Clinical breast examination is costly
9	Clinical breast examination is not necessary in the absence of symptoms
10	Mammogram is important for early detection of breast cancer
11	Mammogram is safe
12	Mammogram is painful
13	Mammogram is time-consuming
14	Mammogram is costly
15	Mammogram increases the risk of breast cancer.
16	Mammogram is not sufficient for detecting cancer
17	Mammogram is not available at nearby healthcare centers

15. If you want to know more about breast cancer and its associated tests, what is your source of information
    a. Scientific websites on the internet
    b. Social media
    c. Friends or family
    d. Private doctor clinic
    e. Healthcare worker
    f. Others

Future practices

1. During the next 12 months, are you planning to conduct early breast examination at a regular rate
    a. Yes
    b. No
    c. I don’t know
2. Which types of early breast examinations will you do
    a. SBE
    b. CBE
    c. MM
3. Which interventions would improve the awareness of women towards early breast examinations of any kind
    a. Increase awareness
    b. Making sure doctors advice women on such issue
    c. Include the tests in the insurance coverage
    d. Presence of symptoms
    e. Encouragement from family
    f. Presence of nearby center
    g. Presence of competent healthcare workers
    h. Easier logistics
    i. I don’t know
    j. Others
4. If you knew that the current practices decrease the risk of cancer, which of the following would you adopt in the next 12 months?
    a. Stop smoking

b. Eating healthy
c. Sleeping for at least 8 hours
d. Playing sports
e. Looking out for my weight
f. Frequently visiting doctors for testing
g. Eating vegetables and fruits
h. Eating moderate amounts of sugar or salt
i. Not stressing
