# Supplementary material for: Exploring barriers to early breast examination and screening among Arab women in the MENA region: A KAP study
Source: Heliyon. 2025 Jan 23;11(3):e42167. doi: 10.1016/j.heliyon.2025.e42167 (PMC11815657; doi:10.1016/j.heliyon.2025.e42167)
Supplement: Multimedia component 2 [file mmc2.pdf]

Supplementary Material

Additional File 2

Exploring barriers to early breast examination and screening among Arab women in the MENA region: A KAP study

Supplementary Table 1: Differences in mean age among participants

Variable	Category	SBE	p-value	CBE	p-value	MM	p-value
Awareness of EBE	Aware	35.90 ± 10.23	<0.001	36.49 ± 10.12	<0.001	37.09 ± 10.04	<0.001
	Unaware	32.88 ± 8.34		33.98 ± 9.88		30.07 ± 9.36	
Practice of EBE	Practicing	37.10 ± 10.27	<0.001	40.21 ± 10.14	<0.001	48.10 ± 6.33	<0.001
	Not practicing	32.51 ± 9.28		32.88 ± 8.71		44.71 ± 5.45	

Supplementary Table 2: Differences in knowledge of risk factors score per participants' characteristics

Variable	Category	Knowledge of risk factors score	p-value
Area of residence	Rural	4.18 ± 2.51	0.372
	Urban	4.36 ± 2.60	
Age	Above 39	4.66 ± 2.61	0.559
	Below 39	4.76 ± 2.61	
Country	Sudan	4.56 ± 2.72	0.046
	Jordan	4.40 ± 2.50	
	Saudi Arabia	4.18 ± 2.67	
	United Arab Emirates	4.34 ± 2.24	
	Lebanon	3.72 ± 2.37	
	Palestine	4.38 ± 2.50	
	Bahrain	4.04 ± 2.80	
	Others	3.95 ± 2.46	
